# Correlation between the Periapical Index and Lesion Volume in Cone-beam Computed Tomography Images

**DOI:** 10.22037/iej.v13i2.15040

**Published:** 2018

**Authors:** Etevaldo Matos Maia Filho, Amanda Martins Calisto, Rudys Rodolfo De Jesus Tavarez, Claudia de Castro Rizzi, Raquel Assed Bezerra Segato, Léa Assed Bezerra da Silva

**Affiliations:** a *Department od Endodontics, Ceuma University, Brazil; *; b * School of Dentistry of Ribeirão Preto at University of São Paulo* *, Brazil*

**Keywords:** Apical Periodontitis, Cone-beam Computed Tomography, Endodontics, Periapical Radiography

## Abstract

**Introduction:**

The study aimed to correlate the Periapical Index (PAI), obtained by way of periapical radiographs, with the volume of chronic periapical lesion, obtained through cone-beam computed tomography (CBCT), in the permanent teeth.

**Methods and Materials:**

Radiographs and CBCT images were selected from 35 single-rooted permanent teeth, with fully formed apices, with a diagnosis of pulp necrosis and chronic apical periodontitis that was radiographically visible. Two independent raters evaluated the radiographs on two separate occasions and classified the periapical lesions in accordance with Ørstavik’s PAI. The periapical lesion volume was calculated in the CBCT images. The correlation between the PAI and the lesion volume was calculated using Spearman’s correlation test.

**Results:**

There was a positive, moderate correlation between the PAI and the volume (*r*_s_=0.596; *P*<0.001) where *r*_s_^2^ is equal to 0.355, showing that only 35% of the PAI variation was dependent upon the variation in periapical lesion volume.

**Conclusion:**

The radiographic evaluation of the periapical lesion does not reflect the lesion’s volumetric characteristics as the volume had a moderate effect on the choice of PAI score.

## Introduction

The alteration in the mineralization and the structure of the bone adjacent to the site of the inflammation forms the basis for the diagnosis of chronic apical periodontitis primarily monitored *via* periapical radiographs [1]. As the image produced in the periapical radiograph in a single sagittal plane represents a three-dimensional structure, the angle of the x-ray beam, overlapping of images and contrast may influence the radiographic interpretation and result in underestimation and flaws in the diagnosis and post-treatment follow-up of the apical periodontitis [2-4].

The periapical index (PAI) is a scoring system for evaluating apical periodontitis *via* radiographs [5], that uses a scale of 1 to 5, ranging from *healthy* to *severe periodontitis with exacerbated features*. The index is based on a study by Brynolf [6] which correlated histological and radiographic findings.

The PAI has been used both in clinical and epidemiological studies [7-11] and frequently the increase in the size of the periapical radiolucency in post-endodontic treatment radiographs is interpreted as a treatment failure, while the reduction or absence indicates the occurrence of the repair process [12]. The PAI is also used in a dichotomized manner for success (PAI 1 and 2) and failure (PAI 3, 4 and 5) [13, 14]. However, it has been well established that the anatomical characteristics of the area adjacent to the periapical lesion may interfere with contrast and hamper the evaluation of periapical tissue in the periapical radiographs [1]. It is known that bone loss in chronic PAs, in order to display visible radiolucency in the images obtained from the periapical radiographs, depends on the density and thickness of the bone cortex and the distance between the lesion and the bone cortex [15, 16].

Recent studies have employed cone-beam computed tomography (CBCT) to increase the accuracy with regard to PA diagnosis [17-19], due to the reliability of the volumetric measurements of periapical lesions when using this resource [20-22]. The advantage of CBCT is its ability to rebuild structures in three orthogonal planes (axial, sagittal and coronal), it being possible to make out the precise periapical location and lesion volume [23]. Accordingly, taking into consideration that the findings using CBCT represent the real condition of periapical bone loss, it is appropriate to establish up to what point the PAI reflects the volumetric status of the periapical lesions.

**Figure 1:**
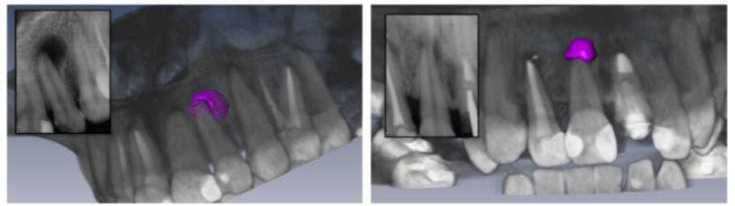
Two examples of periapical lesions reconstructed in 3D using the Amira program, along with the respective radiographic images

Thus, the aim of this study was to correlate the PAI obtained *via* periapical radiographs with the volume of chronic apical periodontitis in permanent teeth, obtained using CBCT.

## Materials and Methods

This study was approved by the research ethics committee at CEUMA University in São Luis, Maranhão, Brazil (29.058 /2013). Research was conducted in full accordance with ethical principles, including the Declaration of Helsinki.

***Selection of the periapical radiographs and the CBCTs***

A total of 35 single-rooted permanent teeth were evaluated (upper and lower incisors and canines and lower premolars), with an indication of radiographic and tomographic examinations unrelated to this study, with a fully formed apex, with a diagnosis of pulp necrosis and radiographically visible chronic apical periodontitis.

***Acquisition of periapical radiographs***

All the periapical radiographs were performed using the bisection technique using digital x-rays (Schick Technologies Inc., Long Island City, NY, USA) and a Seletronic x-ray appliance (Dabi Atlante Indústrias, Médica Odontológica, Ribeirão Preto, São Paulo, Brazil) at 60 kV and 10 mA for 0.2 sec. 

***Criterion for analyzing periapical radiograph images***

Two experienced raters, were calibrated by evaluating 15 radiographs that were not included in this study. The Kappa values ranged from 0.75 to 0.80, demonstrating a strong agreement between the measurements. Moreover, in discordant cases, scores obtained by consensus were included in the final analysis. Cases with different inter-rater scores were reexamined by the two raters and assigned a consensus score which made up the final evaluation. 

The radiographic images were evaluated at separate points in time with an interval of four weeks, interpreted in an environment with subdued lighting by the two independent raters. The radiographs were evaluated on a 2233SW Plus computer monitor (Samsung, Seoul, South Korea) with a screen resolution of 1920×1080 dpi, using the program CDR Dicom for Windows, version 4.1.1.101 (Schick Technologies, Long Island City, NY, USA), it being permitted to use the tools for altering brilliance and contrast, black-and-white inversion and image enlargement. The radiographs were distributed at random to each rater who had to assign a score according to the PAI proposed by Ørstavik *et al.* [5]: *score 1*, normal periapical structure; *score 2*, minor changes in bone structure; *score 3*, change in bone structure with some mineral loss; *score 4*, periodontitis with a well-defined radiolucent area and *score 5*, severe periodontitis with exacerbated features.

***Acquisition of CBCT images***

The CBCT images were obtained using the Next Generation i-CAT tomography machine (Imaging Sciences International, Hatfield, PA, USA). The selected image acquisition protocol was 8 cm diameter × 8 cm high; 0.2 voxel for 8.9 sec, creating tomographic sections of 0.2 mm on three planes (axial, coronal and sagittal). The software i-CAT Vision 1.9.3 was used to reconstruct the three-dimensional images. The data were exported in the Dicom format to the Amira software (v.5.3.3, Visage Imaging Inc., Carlsbad, CA, USA) where the volume of each lesion was calculated. 

The lesion volume calculation, measured in mm^3^, was performed by limiting the area of interest to the apical third by means of the segmentation procedure in the Amira software application. The lesion was demarcated in the axial, coronal and sagittal planes using the “Blow” tool (Gaussian 1, tolerance 35), including radiolucency and excluding the apex of the root. Using the Measurement/Material Statistics tool, the software automatically calculated the periapical lesion volume (Figure 1).

***Statistical analysis***

Spearman’s correlation test was used to find the correlation between PAI scores and lesion volume. The level of significance adopted was 5%. The statistical program employed was SPSS 23.0 (IBM, Armonk, NY, USA).

## Results

A total of 35 teeth, from 21 patients with average age of 36.67±11.21, comprising 14 maxillary central incisors (40.0%), 13 maxillary lateral incisors (37.1%), 2 maxillary canines (5.7%), 2 maxillary premolars (5.7%), 2 mandibular central incisors (5.7%), 1 mandibular lateral incisor (2.9%) and 1 mandibular premolar (2.9%). Table 1 displays the parameters evaluated for each PA index.

**Table 1:** Periapical lesion volume, in mm^3^, observed between the various PAI scores assigned

PAI (N)	Mean (SD)
Score 2 (4)	36.42 (35.82)
Score 3 (9)	55.83 (45.05)
Score 4 (12)	76.10 (55.85)
Score 5 (10)	143.78 (81.21)
Total (35)	70.72 (69.94)

The result of the Spearman correlation was significant (*r*_s_=0.596; *P*<0.001) with the value of r_s_^2^ equal to 0.355.

## Discussion

The PAI is a method based on the correlation between radiographic and histological findings [6], which has, as a limitation, the variation sensitivity between observers in the choice of PAI. To overcome this limitation, in the present study, the radiographs were examined by two raters at two different points in time and the degrees of intra-rater and inter-rater agreement were calculated. 

The correlation between the PAI and the volume of apical periodontitis exhibited a significant result, with r_s_^2^ equal to 0.35, showing that 35% of the PAI variation was dependent upon the volume variation. This means that the “volume” variable was a determining factor in the choice of PAI scores, though not the main factor. Other factors possibly influencing the choice of PAI score included the overlapping of adjacent tissue, the density and thickness of the bone cortex, as well as the distance between the lesion and the cortical bone, as has been demonstrated in other studies [1, 15, 16]. This demonstrates that the reduction in periapical lesions using PAI does not guarantee a volumetric reduction in the lesion, since a lesion with unaltered volume could be classified initially as having one score; but later, influenced by factors such as angle of x-ray beam, overlapping images and contrast [2, 4], have a different score, which may be lower or higher. This outcome corroborates that obtained by Paula-Silva *et al.* [24], who observed that, of the 57 cases (79%) that showed a reduction in or absence of periapical radiolucency evaluated by periapical radiographs, only 25 (35%) saw these results confirmed in a CBCT image, leading to the conclusion that the change in the size of periapical lesions may be inappropriately interpreted in radiographs and may only be properly measured using CBCT. In addition, van der Borden *et al.* [25] concluded that the change in the size of the lesion was different when evaluated by periapical radiography and CBCT, and that the outcome of endodontic treatment determined by way of periapical radiographs may not reflect reality.

PAI is accepted as a valid tool for determining the outcome of endodontic treatment and revealing changes in the extent and severity of the PA. Teeth with decreasing PAI are often regarded as being mended [10, 11]. The results of the present study, however, demonstrated that, despite the existence of correlation, radiographic analysis using PAI may not be telling the whole story of what is happening with the volumetric changes in apical lesions, and that cases of periodontitis with a similar volume may be classified with different periapical index scores, maybe higher, maybe lower, influenced by a variety of factors. Accordingly, the use of radiographs to evaluate PA evolution must be made with caution, as there is a possibility of a false negative or false positive due to the high degree of subjectivity in the evaluation of these lesions using this index. Moreover, there are disadvantages inherent to evaluation *via* periapical radiographs, such as the fact that the image produced in the periapical radiographs in two dimensions and one single sagittal plane represents a three-dimensional structure.

The results of this study have shown that periapical lesion volume has a partial effect on the choice of PAI score and that other factors may exert a strong influence on the radiographic analyses of lesions to the point of masking the method’s accuracy. Other studies have already found low levels of accuracy with periapical radiographs [19, 26, 27], failing to detect as much as 30 to 45% of periapical lesions [28, 29]. So whenever possible, the monitoring of cases of apical periodontitis should be conducted using CBCT, a method capable of analyzing lesion volume with accuracy and precision [30].

## Conclusion

Radiographic evaluation of periapical lesions must be carried out with caution, as it may not reflect the lesion’s volumetric characteristics since, despite being significant, the volume effect was moderated on the choice of Periapical Index.
